# A Bayesian Framework with Dirichlet Priors and Spatial Smoothing for Protein Rotamer Prediction

**DOI:** 10.3390/ijms27062869

**Published:** 2026-03-22

**Authors:** Kamal Al Nasr, Ahmad Jad Allah, Mohammad Alamri, Mohammad Al Sallal

**Affiliations:** 1 Department of Computer Science, Tennessee State University, Nashville, TN 37209, USA; aallah@my.tnstate.edu (A.J.A.); malamri@my.tnstate.edu (M.A.); 2 ACOE Automation, HCA Healthcare, Nashville, TN 37203, USA; msallal@live.com

**Keywords:** protein modeling, amino acid sidechain, sidechain packing, rotamer, rotamer library, backbone-dependent rotamer library, Bayesian probability, Dirichlet priors

## Abstract

Accurate prediction of protein sidechain conformations is a fundamental challenge in structural biology, with diverse applications ranging from protein structure determination to computational drug design. The performance of backbone-dependent rotamer libraries is often limited by discrete binning artifacts and difficulties handling sparse conformational regions. In this work, we present a Bayesian framework for rotamer prediction that addresses these limitations through Dirichlet priors and spatial smoothing. Our approach models rotamer probabilities as continuous functions of backbone dihedral angles, using circular Gaussian convolution, to make the most of statistical strength from neighboring conformations while respecting the periodic nature of angular data. We constructed rotamer libraries through structural clustering of sidechain conformations rather than chi angle binning, ensuring that each rotamer represents a distinct three-dimensional geometry. We evaluated and compared our framework against the state-of-the-art libraries on two independent test sets. Our Dirichlet model achieved chi angle prediction accuracy of 59–60%. Notably, our method produced consistently lower angular errors, an approximate 13% reduction in mean deviation, suggesting that the continuous probability distributions better capture subtle conformational preferences. Further, we explored the incorporation of non-sequential context by including the identity of nearby non-neighboring residues as an example of extensibility of our framework.

## 1. Introduction

**Figure 1:**
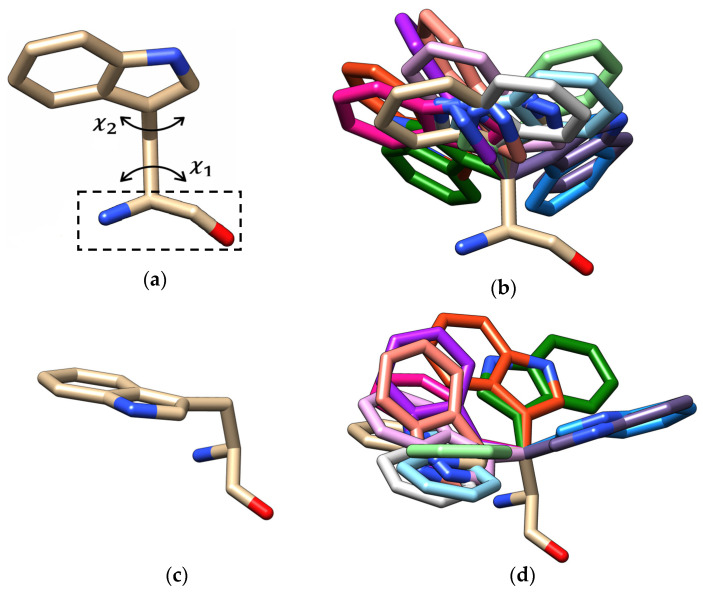
Rotameric states of tryptophan (TRP). (**a**) The two chi angles $\chi_{1}$ (N-Cα-Cβ-Cγ) and $\chi_{2}$ (Cα-Cβ-Cγ-Cδ_1_) that control TRP sidechain orientation. The dashed box indicates the backbone atoms; (**b**) superimposition of a sample of multiple TRP rotamers (shown in different colors) from our dataset, illustrating the discrete conformational states adopted by the sidechain in different occasions; (**c**) the sidechain shown from another orientation; (**d**) the 12 rotamers in (**b**) shown from the orientation in (**c**).

Predicting the three-dimensional (3D) conformation of an amino acid’s sidechain has been a long-standing problem in structural biology with applications in protein structure prediction, homology modeling, and computational drug design [1,2,3,4,5]. An amino acid sidechain typically has preferred rotamers (conformations) defined by their dihedral chi (χ) angles. These specific rotameric/conformational states arise due to both steric and torsional restrictions identified in the 1930s [6] and are determined by the number of χ angles present. For example, glycine (GLY) and alanine (ALA) have no χ angles; they have one possible conformation. In contrast, lysine (LYS) and arginine (ARG) have four χ angles; therefore, they have numerous possible rotameric states. Figure 1 shows an example of 12 random rotameric states of tryptophan (TRP) illustrated using UCSF Chimera v1.19 software [7]. Sidechain rotamer libraries are compilations of rotamer preferences that are represented as probability distributions of possible sidechain conformations that are compiled from high-resolution data, where the probability distribution of each rotamer is based upon the χ angle value(s) of a particular sidechain and the backbone torsion angles phi (φ) and psi (ψ) [1,8,9]. Libraries are broadly classified as backbone-independent, backbone-dependent, or secondary structure-dependent [8,10]. The majority of current rotamer libraries used in protein modeling stem from backbone-dependent libraries. Molecular dynamics simulations have further extended rotamer sampling beyond the static crystallographic ensemble [11,12].

Systematic characterization of rotameric preferences dates to the 1970s [1,4,5,13,14]. In the late 1980s and early 1990s, Dunbrack and Karplus [9] developed a method for defining backbone-dependent rotamer libraries, which they defined by tabulating χ1/χ2 rotamer frequencies as a function of backbone dihedral angles. During the late 1990s, researchers addressed issues like non-rotameric outliers and incorporated more sophisticated statistical methods to handle sparse data [10,15]. Subsequently, Bayesian methods were developed to help regularize rotamer probability estimates within discrete backbone bins [15]. Their approach treated each bin independently, without explicitly modeling the spatial correlation expected in conformational space. In 2011, Shapovalov et al. [16] developed the Dunbrack 2011 rotamer library, derived from an expanded crystallographic dataset and adaptive kernel density estimation. The Dunbrack 2011 library has become the de facto standard and is the basis for many widely used software packages such as MODELLER [2], Rosetta [17], FoldX [18], and SCWRL [19]. Recent approaches integrate Dunbrack rotamer statistics into hybrid pipelines that combine probabilistic rotamer selection with continuous minimization and machine learning based scoring for sidechain and protein structure modeling [20,21,22,23,24,25,26].

Classical backbone-dependent libraries do not explicitly account for a residue’s environment in space (i.e., how it is affected by its neighboring residues) except through φ/ψ angles. Recognizing that neighbor residues can influence rotamers, some research introduced context-dependent rotamer libraries. Bhuyan et al. [27] used a method that incorporates information about the residue(s) that immediately surround each residue of interest. Dicks et al. [28] developed an approach to derive a sequence-dependent library using exhaustive tripeptide simulations. Most recently, Grybauskas et al. [29] used an “on-the-fly” method to generate libraries based upon the structural environment of a residue within a given target protein’s backbone and steric environment. These types of approaches have limitations in terms of their ability to be generally applied as they tend to be either protein-specific or sequence-instance specific.

In contrast to [15], we introduce a framework that incorporates spatial smoothing through circular Gaussian convolution and create continuous probability distributions that explicitly capture the smooth variation in rotamer preferences across backbone space and incorporation of additional structural information. Unlike previous context-based rotamer approaches, our Bayesian framework is designed as a general, population-level probabilistic framework rather than a protein- or sequence-instance specific method. Our framework combines several desirable properties such as proper treatment of angular periodicity through circular convolution and computational efficiency suitable for several applications. In this work, we compare our results directly with the established Dunbrack 2011 library and explore the extensibility and generalization of our framework through the addition of a simple contextual descriptor.

## 2. Results

We evaluated sidechain prediction accuracy of our proposed method, the n-dimensional Dirichlet-smoothed probabilistic model, and compared it against the state-of-the-art Dunbrack 2011 backbone-dependent rotamer library. For both models, predictions were evaluated using two independent test settings. The first test set is a protein level split: Set A. Set A is a set of 100 proteins that was randomly selected from the cleaned original dataset. All residues from these proteins were used for testing. The second test set is an amino acid level split: Set B. Set B is a set of 90K amino acid instances that was randomly selected from the cleaned original dataset. It is worth mentioning that both test sets were excluded from the original dataset and separated from the training set used for our model.

For each test residue, we predicted the most probable rotamer by identifying the rotamer with maximum posterior probability at the residue’s x configuration as in Equation (6). The predicted rotamer was then mapped to its canonical χ angle values $\left({\bar{\chi }}_{1},{\bar{\chi }}_{2},{\bar{\chi }}_{3},{\bar{\chi }}_{4}\right)$ which were computed from the training data and used to generate predicted sidechain conformations. Residues falling outside the defined grid space were excluded from prediction.

We evaluated prediction accuracy using χ angle deviation, which quantifies the angular difference between predicted and observed χ angles. For each residue, we computed the symmetric angular difference for each χ angle:

(1) $$\Delta \chi =min(|\chi_{\mathrm{pred}}-\chi_{\mathrm{true}}|,\;360{}^{\circ}-|\chi_{\mathrm{pred}}-\chi_{\mathrm{true}}|)$$

For amino acids with symmetric sidechains (e.g., PHE, TYR, ASP at $\chi_{2}$; VAL at $\chi_{1}$), we applied an additional symmetry correction:

(2) $$\Delta \chi_{\mathrm{sym}}=min(\Delta \chi ,180{}^{\circ}-\Delta \chi )$$

Following established conventions, we considered that a χ angle is correctly predicted if Δχ≤40°. For residues with multiple χ angles, we used a sequential criterion: $\chi_{1}$ accuracy, $\chi_{1+2}$ accuracy (both $\chi_{1}$ and $\chi_{2}$ correct), $\chi_{1+2+3}$ accuracy, and $\chi_{1+2+3+4}$ accuracy. The overall χ angle accuracy for each amino acid was calculated as [16]:

(3) $$\mathrm{Accuracy}=\frac{N_{1}+N_{12}+N_{123}+N_{1234}}{n_{\chi }\cdot N_{\mathrm{res}}}\times 100\%$$

where $N_{k}$ represents the count of residues with all χ angles up to position k correctly predicted, $n_{\chi }$ is the actual number of χ angles for that amino acid, and $N_{\mathrm{res}}$ is the total number of test residues.

To ensure fair comparison with the Dunbrack 2011 library, we addressed the rotamer definition discrepancy as follows. Our structure-based clustering produced variable rotameric states across all amino acids, whereas the Dunbrack 2011 library defines three canonical rotamer states for each χ angle. For evaluation purposes, both methods were assessed directly against the experimentally observed χ angles in the test sets, which were treated as ground truth. Neither method’s internal rotamer definitions were privileged; instead, both were evaluated solely on their ability to predict the actual χ angles in test structures.

The Dunbrack 2011 rotamer library employs 10° bins in φ/ψ space, and our Dirichlet framework used the same binning scheme to ensure direct comparability. For each test residue, both methods queried the identical (φ, ψ) bin derived from the experimentally observed backbone angles. We did not apply backbone interpolation; both methods used discrete 10° bins as defined in the original library. This ensured that differences in predictive performance resulted from modeling strategy rather than backbone discretization or smoothing procedures.

In addition, we computed MADχ, defined as the mean absolute angular deviation (in degrees), across all predictions to provide continuous measures of prediction quality. All evaluations were performed separately for each amino acid type, and results were aggregated to assess both models’ performance.

### 2.1. Performance of Dunbrack Library

We evaluated the Dunbrack 2011 backbone-dependent rotamer library [16] on sets A and B. In both cases, predictions were generated by selecting the most probable rotamer corresponding to the amino acid type and backbone φ/ψ bin, and χ angle accuracy was evaluated using Equation (3). The results are reported in Table 1. Overall, Dunbrack’s method achieved a χ angle accuracy of 59.36% on Set A and 60.49% on Set B, with mean angular deviations (MADχ) of 47.21° and 45.68°, respectively. The close agreement between these values indicates that the Dunbrack 2011 performance is consistent across structure-level and residue-level evaluation regimes, with only a modest improvement observed in the larger, residue-balanced test set, Set B.

To carefully assess the Dirichlet framework’s contribution while eliminating dataset-related confounds, we developed an in-house implementation of the Dunbrack 2011 using our dataset for training/testing. This design isolates pure methodological differences by comparing methods trained on identical data.

Our in-house implementation of the Dunbrack 2011 reproduced the original library’s performance with comparable accuracy across both test sets (Test Set A: 58.89% vs. 59.36% accuracy, 47.81° vs. 47.21° MADχ; Test Set B: 60.12% vs. 60.49%, 46.41° vs. 45.68°). Maximum deviations of 0.47% in accuracy and 0.73° in MADχ confirm both implementation correctness and indicate that dataset evolution alone does not materially account for the observed performance differences. This validation establishes a fair baseline for methodological comparison. Therefore, given the negligible deviation between the original and in-house implementations, subsequent comparisons are presented using the published Dunbrack 2011 library for consistency with prior literature.

Performance varied substantially across amino acid types and correlated with sidechain complexity. Amino acids with simple, constrained sidechains exhibited the highest accuracy: VAL (86.35% and 86.61%), THR (79.39% and 81.47%), and ILE (76.70% and 77.92%) on the two test sets, respectively. These residues have limited rotameric degrees of freedom (one or two χ angles), and their conformational preferences are strongly dictated by steric constraints.

In contrast, amino acids with longer, more flexible sidechains showed markedly lower performance. ARG, which has four χ angles and substantial conformational freedom, achieved only 35.46% and 35.18% accuracy with MADχ near 49.3° on both test sets. LYS (39.58% and 41.00%) and MET (37.84% and 41.23%) exhibited similar difficulties, suggesting that backbone geometry alone provides insufficient information to accurately predict distal sidechain conformations for highly flexible residues. GLN (37.41% and 37.68%) also showed poor performance despite having three χ angles, likely reflecting its polar nature and sensitivity to local environments not captured by backbone-dependent models.

Amino acids with two χ angles displayed intermediate performance, with considerable variation. PRO achieved 75.17% and 78.87% accuracy, benefiting from its unique cyclic structure that severely restricts conformational space. LEU (67.52% and 68.38%), ASP (65.01% and 66.42%), and PHE (69.62% and 72.05%) showed good performance, while ASN (53.27% and 58.00%) and HIS (50.31% and 50.81%) were less accurately predicted, possibly due to their ability to form diverse hydrogen-bonding interactions that influence rotamer preferences outside backbone geometry.

Notably, performance consistency between the two test sets was high, with most amino acids showing less than 2% variation in accuracy. This consistency validates the robustness of the Dunbrack 2011 library across different sampling strategies and suggests that the observed performance levels represent genuine limitations of a sole backbone prediction rather than statistical artifacts of the test sets.

### 2.2. Performance of the Dirichlet Bayesian Model

Our Dirichlet-based Bayesian model was evaluated under two distinct contextual definitions: using backbone angles alone (φ, ψ) (see Table 2) and incorporating the identity of the non-sequential spatial contact derived from nearest tertiary neighbors (e.g., the biochemical class of the closest j residue within a sphere of 5.5 Å radius) (see Table 3). The nearest neighbor is categorized using its biochemical property. To reduce categorical complexity while maintaining chemical properties, amino acid identity of contact residue is mapped to two coarse biochemical classes, either hydrophobic or non-hydrophobic. This approach tests whether local spatial information beyond immediate neighbors can improve rotamer prediction accuracy.

#### 2.2.1. Backbone-Only Context

Using backbone geometry alone, the Dirichlet model achieved overall χ angle accuracy of 59.11% on Set A and 60.05% on Set B, with MADχ of 41.22° and 39.86°, respectively (see Table 2). These results are remarkably similar to the Dunbrack 2011 performance, suggesting that both methods capture comparable information from backbone dihedral angles. However, the Dirichlet model demonstrated substantially lower MADχ (approximately 6° improvement on Set A and 5.8° improvement on Set B), indicating that when predictions are incorrect, they tend to be closer to the true values compared to the Dunbrack 2011 library.

The Dirichlet model replicated the general performance hierarchy observed with the Dunbrack 2011 method. VAL achieved the highest accuracy (86.41% and 86.88%), followed by THR (79.32% and 80.86%), PRO (76.41% and 79.83%), and ILE (76.29% and 77.98%). These simple, conformationally restricted residues benefit from the model’s ability to learn sharp probability distributions concentrated around dominant rotameric states.

Long chain flexible residues remained challenging: ARG (34.96% and 34.52%), GLN (38.40% and 38.54%), and LYS (40.13% and 40.73%) showed accuracy below 41%. MET (40.40% and 40.91%) also exhibited poor performance despite its hydrophobic nature, likely due to the three rotatable bonds allowing extensive conformational sampling. MADχ for these residues ranged from 42° to 60°, with ARG showing the largest deviations (49.28° and 49.70°), reflecting the difficulty in predicting far χ angles in extended sidechains.

Aromatic residues showed good performance: PHE (69.23% and 71.60%), TYR (69.67% and 69.24%), and TRP (50.91% and 52.32%). The bulky aromatic rings impose significant steric constraints that the model effectively captures through the smoothed probability distributions. The relatively lower performance of TRP likely reflects its unique bicyclic indole system and capacity for diverse stacking interactions.

#### 2.2.2. Non-Sequential Context

Incorporating the identity of the closest non-neighboring residue to backbone geometry as additional context produced mixed results. On Set A, overall accuracy improved marginally to 59.36% (from 59.11%), while Set B showed a slightly larger increase to 60.32% (from 60.05%). MADχ decreased modestly to 41.04° and 39.82°, respectively (see Table 3).

**Table 3:** Dirichlet method performance on backbone + non-sequential contact.

AA	Set A		Set B	
	Acc. %	MADχ	Acc. %	MADχ
ARG	35.13	49.75	33.96	50.70
ASN	53.25	54.90	56.74	46.45
ASP	65.50	33.79	65.90	32.95
CYS	68.01	42.31	68.30	42.24
GLN	37.35	58.98	39.36	54.50
GLU	41.09	43.59	41.35	42.95
HIS	48.60	61.52	49.46	61.72
ILE	76.66	27.68	77.60	27.19
LEU	67.15	32.25	69.63	30.84
LYS	40.69	42.14	40.75	41.52
MET	41.11	59.27	41.03	60.96
PHE	69.30	28.76	71.09	27.05
PRO	76.68	20.89	79.69	16.59
SER	59.77	52.99	60.28	53.15
THR	80.03	29.67	80.98	28.06
TRP	51.83	58.46	52.48	58.06
TYR	69.86	27.77	69.71	28.49
VAL	86.45	14.05	87.38	13.32
Average	59.36	41.04	60.32	39.82

The impact of additional non-sequential context varied substantially across amino acid types. Several residues showed improvements: ASN increased from 52.21% to 53.25% on Set A, LEU from 67.60% to 69.63% on Set B, SER from 59.08% to 59.77% on Set A and from 58.54% to 60.28% on Set B, and VAL showed slight gains on Set B (86.88% to 87.38%). These improvements suggest that local sequence environment influences rotamer preferences for certain residues, particularly those capable of forming specific interactions with nearby sidechains.

Extending the conditioning space to include the biochemical class of the nearest non-sequential contact residue led to modest but systematic improvements across both evaluation sets. Although the absolute numerical gains are limited, they are observed across a wide range of amino acids. Improved performance was observed for 15 amino acids (Set A) and 12 amino acids (Set B) out of 18 amino acids. Residues whose conformations are particularly sensitive to local packing interactions such as LEU, ILE, THR, PRO, and SER exhibited the most consistent improvements.

These findings support the established principle that backbone geometry plays a dominant role in determining rotamer conformations, with local environmental factors exerting a subtle, secondary influence [30,31,32]. The limited magnitude of improvement likely reflects both this backbone dominance and the coarse nature of the binary hydrophobic/non-hydrophobic classification. Many environmental influences are partially correlated with backbone conformation or require richer descriptors, such as solvent accessibility, contact density, or electrostatics, to exert measurable effects. Thus, substantial gains should not be expected from a single binary contextual variable. Further, despite the marginal improvement of the context, which was defined at a coarse biochemical level to test extensibility, this highlights a key architectural advantage of our model that enables seamless integration of additional structural or environmental without altering the underlying inference mechanism. Furthermore, the consistent reduction in MADχ suggests that the model captures finer-grained conformational preferences, even when overall rotamer prediction accuracy remains similar.

#### 2.2.3. Mean Angular Deviation Analysis

To assess whether observed differences in MADχ represent genuine methodological improvements versus sampling artifacts, we performed paired two-tailed *t*-tests on per-residue angular deviations. For each test residue, both methods predicted the same structure, yielding paired observations (δDunbrack, δDirichlet). Because predictions are made at the residue level, statistical testing was conducted on paired per-residue angular deviations rather than aggregated protein-level summaries, providing higher resolution and statistical power. The paired design eliminates inter-residue variability, which is large but irrelevant to comparing methods, and directly tests whether mean (δDirichlet—δDunbrack) differs significantly from zero.

We report mean differences (Δ), 95% confidence intervals, t-statistics, *p*-values, and Cohen’s d effect sizes (interpreted as small > 0.2, medium > 0.5, large > 0.8). Statistical significance was assessed at α = 0.05 for overall comparisons and α = 0.0028 for per-amino acid tests (Bonferroni correction: 0.05/18). With sample sizes exceeding 18,000 residues, the Central Limit Theorem ensures normality of mean differences, validating parametric testing. Bootstrap resampling (1000 iterations) confirmed robustness of confidence intervals. McNemar’s test assessed categorical accuracy differences.

Paired *t*-tests established significant improvement in angular precision for the Backbone-Only Context Dirichlet framework (Table 4). On Test Set A (*n* = 18,991 residues), MADχ decreased from 47.21° (Dunbrack 2011) to 41.22° (Dirichlet), a reduction of 6.0° (95% CI: [5.2°, 6.8°], t = 14.73, *p* < 10^−45^, Cohen’s d = 0.31). Test Set B (*n* = 90,000) showed consistent results: 45.68°→39.86°, Δ = 5.8° (95% CI: [5.5°, 6.1°], t = 33.04, *p* < 10^−220^, d = 0.29). The medium effect size indicates not only statistical significance but practical relevance; a 12–14% reduction in angular error is meaningful for structure refinement and modeling applications.

#### 2.2.4. Parameter Sensitivity Analysis

The Dirichlet concentration parameter alpha (α) directly controls shrinkage toward the rotamer prior, and the kernel parameters (radius, sigma (σ)) determine the degree of spatial interpolation across the context grid. α = 40 corresponds to approximately two pseudo-counts per canonical χ state. To assess the impact of these parameters on the accuracy of our model, we evaluated the robustness of the backbone + non-sequential context model on Set A across a range of α values and smoothing settings (Table 5). Varying α from 20 to 100 while fixing radius = (1, 1, 1) and σ = (1, 1, 1) yielded indistinguishable performance (differences < 0.01%) in mean χ-angle accuracy (59.36%) and mean angular deviation (MADχ = 41.04°), indicating that predictive performance is effectively invariant to α across this range. Adjusting the spatial smoothing strength produced only marginal changes: for example, increasing σ to 1.5–2.0 (radius = (1, 1, 1), α = 40) increased χ-angle accuracy slightly to 59.43% with MADχ ≈ 40.89°, while increasing the smoothing radius to (2, 2, 2) yielded 59.42% accuracy and MADχ = 40.88°. Furthermore, removing neighborhood smoothing (radius = (0, 0, 0), σ = (1, 1, 1), α = 40) produced comparable performance (59.38%, MADχ = 40.94°). Collectively, these results show that the proposed framework is robust to the precise choice of α and to moderate variations in the smoothing kernel, supporting the use of fixed theoretically motivated parameters in the main evaluation.

We did not adopt the strongest smoothing settings despite minor gains, as they broaden interpolation and reduce spatial locality. On the other hand, we instead report the conservative baseline (radius = 1, σ = 1, α = 40) to avoid over-smoothing across distinct conformational regions.

Lastly, our fixed-bandwidth Bayesian approach differs from Dunbrack 2011’s adaptive kernel density estimation. Unlike Dunbrack 2011’s adaptive bandwidth approach that handles sparsity through variable kernel widths, our framework addresses sparsity via the Dirichlet prior while maintaining fixed spatial smoothing, separating categorical uncertainty from spatial correlation structure. Dunbrack 2011 adjusts bandwidth inversely with data density, while we separate spatial smoothing (fixed Gaussian kernel) from sparsity handling (Dirichlet prior). Both approaches navigate the bias-variance tradeoff through different mechanisms, achieving similar categorical accuracy (~59–60%), while our approach provides superior angular precision (12–14% MADχ reduction).

## 3. Discussion

Direct comparison between the Dirichlet model (backbone only context) and Dunbrack’s rotamer library reveals remarkably similar overall performance. On Set A, both methods achieved nearly identical χ angle accuracies (59.11% for Dirichlet vs. 59.36% for Dunbrack, a difference of only 0.25%). On Set B, the difference was similarly negligible (60.05% vs. 60.49%, a 0.44% difference). This close agreement suggests that both approaches effectively capture the fundamental relationship between backbone geometry and rotamer preferences, despite employing fundamentally different methodologies.

However, the Dirichlet model demonstrated a clear advantage in prediction precision. MADχ were consistently lower: 41.22° versus 47.21° on Set A (5.99° improvement, 12.7% reduction) and 39.86° versus 45.68° on Set B (5.82° improvement, 12.7% reduction). This systematic reduction in angular error indicates that the Bayesian smoothing approach produces more refined probability distributions that, when incorrect, tend to select rotamers closer to the true conformation. The circular Gaussian kernel smoothing likely contributes by creating gradual probability transitions across conformational space rather than sharp bin boundaries, resulting in more physically reasonable predictions.

Performance patterns across amino acid types were highly correlated between the two methods with Pearson correlation r > 0.98 for both test sets. This confirms that the same intrinsic structural factors govern prediction difficulty regardless of methodology. Both methods excel at predicting simple, sterically constrained residues (VAL, THR, ILE, PRO) and struggle with long, flexible sidechains (ARG, LYS, GLN, MET). This consistency suggests that fundamental limitations exist in predicting rotamers from backbone geometry alone, particularly for residues whose χ angles are influenced by medium-range interactions, solvent exposure, and functional constraints not encoded in local backbone structure.

Certain amino acids showed modest performance differences favoring one method over the other. On Set B, Dunbrack 2011 library outperformed the Dirichlet model for ASN (58.00% vs. 56.53%), PHE (72.05% vs. 71.60%), and PRO (78.87% vs. 79.83%). Conversely, the Dirichlet model showed advantages for ASP (65.52% vs. 66.42% on Set B), LEU (67.60% vs. 68.38% for Dunbrack on Set B, but 69.63% for Dirichlet with backbone + context), and maintained near-parity for most other residues. These differences, typically ranging from 0.5% to 2%, likely reflect subtle variations in how the methods handle data sparsity and boundary regions of conformational space.

Overall, these results demonstrate that the proposed Dirichlet framework constitutes a flexible and extensible generalization of classical rotamer libraries, achieving comparable or improved accuracy while enabling principled integration of structural context. Further, from a practical standpoint, both methods achieve reasonable accuracy for common structural modeling applications. The Dirichlet model’s advantage in angular precision may be particularly valuable for applications requiring quantitative accuracy, such as refinement protocols or energy function development. However, the similar overall accuracies suggest that fundamental advances in rotamer prediction will likely require incorporation of additional physical or evolutionary information beyond local backbone geometry and simple sequence features.

## 4. Materials and Methods

### 4.1. Dataset and Preprocessing

We analyzed a comprehensive dataset of protein structures downloaded from PISCES server [33]. The dataset (cullpdb_pc25.0_res0.0-3.0_len40-10000_R0.25_Xray+Nmr+EM_d2025_09_02_chains15330) contains 15,330 chains. We could read 13,276 chains consisting of approximately 3.44 M residues that span multiple instances of amino acid types. We dropped any residues missing any of the backbone or sidechain atoms. After cleaning, the dataset consisted of approximately 2.9 M residues. To ensure generalizability, we partitioned the dataset into three lists. The first list was used for calculating our rotamer library, the training set. The second list, Set A, contains 100 randomly selected chains consisting of about 19,000 amino acids. The third list, Set B, contains 90,000 randomly selected amino acid entries/instances, comprising 5000 instances for each amino acid (excluding GLY and ALA). Sets A and B were used for testing. There is no overlap between any of the three sets.

### 4.2. Rotamer Clustering

In this research, amino acids’ sidechain conformations were represented using rotamer states derived directly from structural data. For each amino acid, observed sidechain conformations were clustered in dihedral-angle space using a three-phase root-mean-square deviation (RMSD)-based algorithm designed to identify geometrically coherent rotamer basins without predefining the number of clusters. Unlike k-means, the number of clusters is not specified in advance, and unlike kernel density-based mean shift, the method does not rely on bandwidth-dependent density estimation. Instead, clusters are defined by physically interpretable hard RMSD thresholds applied to reconstructed sidechain geometries.

Each amino acid was processed independently using a residue-specific RMSD cutoff (*t*Å), chosen according to conformational flexibility (Table 6) such that the conformations of a cluster are within (tÅ) to the centroid. The algorithm consists of sequential initialization, Lloyd-style iterative refinement, and hierarchical merging. In phase one (initialization), rotamers were sequentially processed. If a rotamer’s RMSD to an existing cluster centroid was within the residue-specific threshold tÅ, it was added to that cluster, and the centroid conformation was recalculated. Otherwise, it seeded a new cluster. This phase produces an initial adaptive partition without requiring prior specification of cluster count. In phase two (refinement), iterative reassignment was applied. Each conformation was tested against all cluster centroids and moved to the nearest cluster if a closer assignment existed within threshold tÅ. Both affected centroids are recalculated after each move. This process was repeated until convergence (no reassignments occurred). This phase is comparable to the Lloyd’s algorithm refinement step in k-means clustering, ensuring each conformation occupied its optimal cluster assignment. In phase three (merging), the final clusters are produced. This phase identifies clusters with inter-centroid RMSD below 0.7 × tÅ and combines them into a larger cluster with updated centroid, addressing potential over-fragmentation from the initialization phase. The 0.7 factor was empirically selected to merge near-duplicate centroids while preserving distinct basins.

Although phase one (initialization) processes conformations sequentially, the subsequent refinement and merging steps substantially reduce potential order dependence. To assess stability, clustering was repeated using multiple randomized processing orders. Final cluster counts and centroid geometries were highly consistent across runs, with negligible variation in cluster number and sub-Å differences in centroid RMSD. These results indicate convergence to stable cluster configurations under the specified RMSD thresholds.

**Figure 2:**
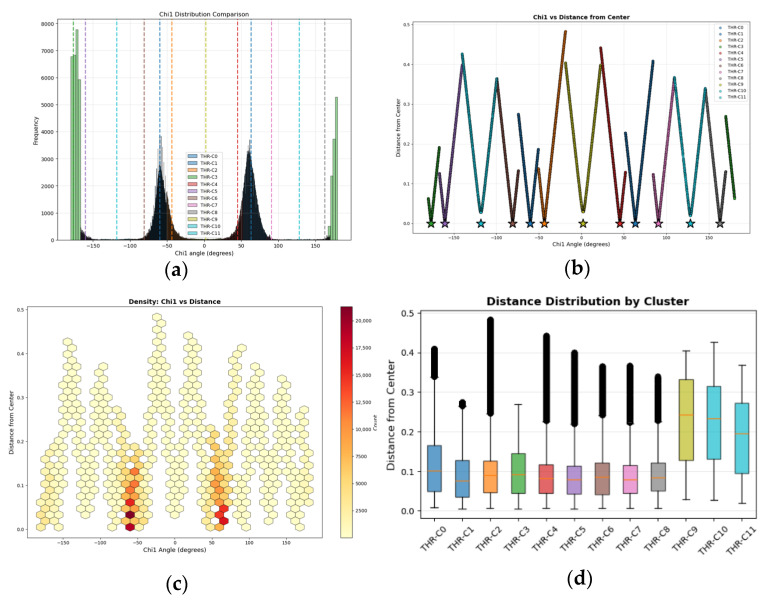
Conformational analysis of the 12 rotamer states for Threonine (THR). (**a**) Chi1 ($\chi_{1}$) angle distribution comparison across different THR 12 clusters. Each dotted line represents the representative rotamer (center) of the cluster; (**b**) scatter plot of $\chi_{1}$ versus distance (RMSD) from cluster center, showing the relationship between angular deviation and conformational similarity. Stars denote cluster centers at zero distance; (**c**) density heatmap highlighting regions of high conformational sampling, with warmer colors indicating more frequently observed $\chi_{1}$-distance combinations; (**d**) box plots comparing the spread of distances within each cluster, demonstrating varying degrees of conformational homogeneity. The solid line inside the box is the mean distance between the center and the rest of rotamer conformations belong to the cluster.

Each cluster was represented by its center, which serves as the canonical rotamer for that cluster. This approach ensures that rotamer definitions reflect observed structural variability while avoiding arbitrary discretization of χ angle space. The resulting rotamer sets are amino acid specific and structurally defined, comparable to Dunbrack style rotamers but derived directly from clustering rather than fixed angular bins. Figure 2 shows conformational analysis of the 12 rotamer states for Threonine (THR). Looking at the distribution of the 12 distinct clusters, we see that THR adopts well-defined rotameric states that are clearly separated in $\chi_{1}$ angle space.

RMSD thresholds (tÅ) for rotamer clustering were assigned based on conformational flexibility, ranging from 0.30 Å for rigid residues to 0.90 Å for highly flexible chains (Table 6). Tight thresholds (0.30–0.40 Å) were applied to short side chains (VAL, THR, SER, CYS), β-branched residues (ILE, LEU), aromatic residues (PHE, TYR), and the conformationally restricted PRO, where limited rotational degrees of freedom define well-separated rotameric states. These thresholds produced cluster counts consistent with expected conformational diversity (i.e., 5–12 clusters for simple residues and 301–459 for aromatics with multiple $\chi_{1}$ orientations). Long, flexible sidechains with three or more χ angles (LYS, ARG, GLN, GLU, MET) received looser thresholds (0.70–0.90 Å) to accommodate their extensive conformational heterogeneity and, as a result, they produced substantial cluster counts (i.e., 123–1061) reflecting genuine structural variation. Intermediate thresholds addressed unique cases: HIS (0.50 Å) for imidazole orientational flexibility, and ILE (0.60 Å) for dual methyl branching effects. This adaptive strategy produced structurally coherent clusters with low mean internal RMSDs. For instance, the internal RMSD is below 0.1 Å for most of the clusters of THR (see Figure 2d).

### 4.3. Generalization of Backbone-Dependent Rotamer Libraries

The framework presented in this work is a non-parametric, context-conditioned Dirichlet-smoothed rotamer probability model, using Gaussian circular kernel density estimation in backbone dihedral space, with probabilistic interpolation in sparsely sampled regions. It can be viewed as a strict generalization of classical backbone-dependent rotamer libraries, including the Dunbrack 2011 formulation. Therefore, in the simplest case where the context space is restricted to backbone φ/ψ angles, neighborhood smoothing is applied only in these two dimensions, and the Dirichlet prior is chosen to be weak and uniform, the model reduces to a smoothed backbone-dependent rotamer frequency table closely analogous to the Dunbrack library.

The proposed framework removes the intrinsic limitation to two-dimensional backbone conditioning. By formulating rotamer probability estimation as Bayesian inference with a Dirichlet prior over multinomial outcomes, the model naturally supports arbitrary numbers of contextual variables, including additional continuous torsions, discretized environmental descriptors, or previously predicted sidechain angles. Smoothing is performed locally and independently along each contextual axis, preserving periodicity where appropriate, and enabling robust interpolation in sparsely sampled regions of high-dimensional space.

This generalization allows hierarchical or sequential conditioning schemes (e.g., predicting $\chi_{1}$ from backbone, followed by $\chi_{2}$ conditioned on backbone and predicted $\chi_{1}$) to be expressed within a single probabilistic framework. Importantly, all probability estimates remain normalized, non-degenerate, and statistically interpretable regardless of dimensionality, without requiring modified heuristics or reparameterization.

As a result, the Dirichlet–neighbor model incorporates traditional backbone-dependent rotamer libraries as a low-dimensional special case while providing a flexible foundation for modern sidechain modeling pipelines that integrate richer structural or learned context.

### 4.4. Probabilistic Framework

In this work, we construct a context and backbone-dependent sidechain rotamer library using a probabilistic framework that combines Dirichlet posterior estimation with local neighborhood smoothing in torsional space. The model estimates conditional probabilities of discrete sidechain rotamers given backbone and additional structural variables, while remaining robust to sparse sampling and missing bins. Let $r\in R_{a}$ denote a rotamer of amino acid a, and let $x=(x_{1},x_{2},\ldots ,x_{d})$ represent a discretized context vector (e.g., backbone φ/ψ angles, additional torsions, or other features binned onto regular grids). Our estimate for a specific amino acid is given by $P\left(r|x\right)$.

#### 4.4.1. Discretization of Conformational Space

Continuous variables such as backbone torsion angles φ/ψ are discretized into uniform 10-degree bins spanning the full angular range (−180° to +180°) to create a structured grid for probability estimation while preserving the periodic nature of angular data. Each residue instance is assigned to a unique multi-dimensional bin $x=(x_{1},x_{2},\ldots ,x_{d}),$ where each dimension corresponds to one contextual variable. The framework supports any number of context variables in addition to the two backbone geometry features φ/ψ. If φ/ψ are the only two variables to study, x=(φ,ψ). For each amino acid type, we collected counts $C_{r}(x)$ representing the number of observations of rotamer r in bin x.

#### 4.4.2. Local Neighbor Smoothing in Context Space

Our Bayesian framework estimates rotamer probabilities as a function of local context. For each amino acid type, we constructed multi-dimensional count tensors $C_{r}(x)$ representing the frequency of observing rotamer r at each contextual configuration. Direct estimation, ${\tilde{C}}_{r}(x)$, of P(r∣x) from raw counts leads to possible sparsity, especially in higher-dimensional contexts. To address data sparsity and leverage the spatial correlation of rotamer preferences across similar contextual conformations, we applied local smoothing over neighboring bins using a separable Gaussian kernel in each dimension. For each dimension i, a one-dimensional Gaussian kernel $K_{i}(\Delta x_{i})$ with radius $R_{i}$ and standard deviation $\sigma_{i}$ is defined. Then, the smoothed count for rotamer r at bin x is given by:

(4) $${\tilde{C}}_{r}(x)=\sum_{y\in N(x)}\left(\prod_{i=1}^{d}K_{i}(y_{i}-x_{i})\right)C_{r}(y)$$

where N(x) denotes the neighborhood defined by the kernel radii in each dimension. In our framework, circular convolution ensures appropriate handling of the periodic boundary conditions inherent to angular data. This procedure propagates statistical support across nearby bins while preserving locality in conformational space.

#### 4.4.3. Dirichlet Posterior Estimation

To convert smoothed counts into probabilities, we apply Bayesian inference with a Dirichlet prior to estimate rotamer probabilities. For each bin x, the posterior probability of observing rotamer r is calculated as:

(5) $$P(r|x)=\frac{{\tilde{C}}_{r}(x)+\alpha \;\pi_{r}}{\sum_{r^{\prime }\in R_{a}}{\tilde{C}}_{r^{\prime }}(x)+\alpha }$$

where α is a concentration (pseudo-count) parameter controlling the strength of the prior and $\pi_{r}$ is the prior probability of rotamer r (i.e., uniform distribution across all rotamers for each amino acid). Unless otherwise specified, $\pi_{r}$ is taken as a uniform prior over rotamers of the given amino acid. This formulation ensures non-zero probabilities in sparsely sampled or empty bins and yields a well-defined posterior distribution for every context. Parameter selection and sensitivity analysis are detailed in Results section. Briefly, we selected α = 40 (Dirichlet concentration), radius = 1 (smoothing neighborhood), and σ = 1.0 (Gaussian width) as mid-range values providing modest regularization.

#### 4.4.4. Handling Missing or Sparse Bins

To balance reliability to observed data with effective smoothing, we implement a hybrid strategy. For bins with sufficient observed data, the posterior is dominated by raw local counts. For bins with few or no observations, the model smoothly interpolates probabilities from neighboring bins and the Dirichlet prior. This approach preserves the empirical distribution where data are abundant while leveraging the smoothing mechanism to make informed predictions in sparse regions of the conformational space. An optional “fill-missing-only” strategy is employed in some experiments, in which raw (unsmoothed) counts $C_{r}$ are used for bins with observations, while smoothed estimates ${\tilde{C}}_{r}$ are used exclusively for empty bins.

To ensure consistent coverage across all amino acid types, we establish a global grid spanning all unique x bins present in the cleaned dataset. This global grid prevents out-of-grid prediction failures that could arise when test cases occupy backbone configurations absent from the training data for specific amino acids.

#### 4.4.5. Rotamer Prediction and χ-Angle Assignment

For each test residue, we predict the most probable rotamer by identifying the rotamer with maximum posterior probability at the residue’s x configuration as:

(6) $$\hat{r}\left(x\right)=\arg\underset{r}{\max}\;P\left(r|x\right).$$

The predicted rotamer is then mapped to its canonical χ angle values $\left({\bar{\chi }}_{1},{\bar{\chi }}_{2},{\bar{\chi }}_{3},{\bar{\chi }}_{4}\right)$ which are computed from the training data and used to generate predicted sidechain conformations. Residues falling outside the defined grid space are excluded from prediction.

Because smoothing and normalization are performed independently for each amino acid, the method scales linearly with amino acid type and supports incremental or memory-efficient construction. Further, our framework naturally extends to any arbitrary numbers of contextual variables (n-dimensional); recall that the simplest model that can be used is with backbone geometry (φ/ψ) only.

### 4.5. Comparison with the Dunbrack 2011 Libraries

The widely used backbone-dependent rotamer libraries developed by Shapovalov et al. (Dunbrack 2011) represent a foundational contribution to protein sidechain modeling [16]. Both the Dunbrack 2011 library and this work aim to estimate conditional rotamer probabilities as a function of local backbone conformation; however, the underlying statistical assumptions, smoothing strategies, and extensibility differ substantially.

#### 4.5.1. Statistical Framework

The Dunbrack 2011 library model P(r∣φ,ψ) uses a parametric mixture framework, in which rotamer frequencies are estimated within fixed backbone bins and smoothed using continuous kernel density estimation (KDE) over the Ramachandran space. Probability mass is redistributed across bins using backbone-dependent smoothing functions derived from observed densities.

In contrast, our approach formulates rotamer probability estimation as a Dirichlet–multinomial posterior inference problem. Raw or smoothed counts are treated as observations from a multinomial distribution, with a Dirichlet prior enforcing regularization. This yields closed-form posterior probabilities with explicit control over the strength of prior information via a single concentration parameter. The probabilistic interpretation is fully Bayesian and remains well-defined even in bins with zero observations.

#### 4.5.2. Smoothing Strategy

The proposed Dirichlet framework employs a fundamentally different smoothing architecture than the Dunbrack 2011 adaptive kernel density estimation approach; Dunbrack’s method applies non-parametric von Mises kernel weighting dynamically at query time computing, using Equation (1) [16] and summing contributions from all training instances/data points with adaptive bandwidth κ. Our approach performs spatial smoothing as a preprocessing step on a discrete grid using separable circular Gaussian convolution ${\tilde{C}}_{r}(x)$ followed by Bayesian inference with Dirichlet priors to obtain P(r∣x).

This architectural distinction (i.e., parametric grid with pre-computed smoothing versus instance-based density estimation) fundamentally alters the computational profile (i.e., O(1) lookup versus O(N) per-query summation), introduces explicit Bayesian regularization through the concentration parameter α that provides interpretable control over prior strength in sparse regions, and trades the adaptive bandwidth flexibility of KDE for computational efficiency and direct probabilistic interpretation. The von Mises kernel’s natural treatment of circular periodicity is replaced by circular convolution with Gaussian kernels and fixed global bandwidth, sacrificing local adaptivity for mathematical tractability and pre-computability.

#### 4.5.3. Treatment of Sparsity and Empty Bins

A central challenge in rotamer libraries is sparse sampling in backbone or higher-dimensional context space. Dunbrack’s method addresses sparsity by borrowing statistical strength globally through KDE smoothing in φ/ψ space, effectively assuming smooth variation in rotamer probabilities over the Ramachandran map.

By contrast, our method uses explicit local neighborhood smoothing via circular Gaussian convolution in each contextual dimension, followed by Dirichlet regularization. This separation of smoothing and normalization allows local interpolation from neighboring bins when data are sparse and produces stable probability estimates. Importantly, Dirichlet posterior ensures that probabilities always sum to one and remain non-zero, even in unobserved regions of conformational space.

#### 4.5.4. Dimensionality and Model Extensibility

The Dunbrack 2011 library is fundamentally two-dimensional, conditioned on backbone ϕ and ψ, with extensions handled implicitly through separate libraries or secondary structure classification. In contrast, the proposed model is natively n-dimensional. Any number of continuous or periodic contextual variables can be incorporated by discretizing each variable, applying separable neighbor smoothing, and performing Dirichlet posterior normalization. This enables direct modeling of higher-order dependencies (e.g., $\varphi ,\psi ,\chi_{1}^{pred}$ or contextual neighborhood) within a single unified probabilistic framework.

#### 4.5.5. Practical Implications

While Dunbrack 2011 library remains the de facto for backbone-dependent sidechain modeling and is highly effective in established sidechain packing pipelines, our approach offers complementary advantages such as robustness in sparsely sampled regions, natural extensibility to higher-dimensional contexts, and compatibility with modern hybrid and machine learning based pipelines that require probabilistic conditioning on multiple continuous features.

## 5. Conclusions

In this work, we developed and validated a Bayesian framework for protein sidechain rotamer prediction that leverages Dirichlet priors and Gaussian spatial smoothing to estimate rotamer probabilities from backbone geometry. Our approach addresses the challenge of data sparsity in conformational space. When evaluated on two independent test sets, the Dirichlet model achieved performance comparable to the state-of-the-art Dunbrack 2011 rotamer library, with an overall χ angle accuracy of approximately 59–60%.

Across two independent test sets, the Dirichlet model achieved χ angle accuracy comparable to those of the Dunbrack 2011 library while consistently producing lower angular deviations. This reduction in χ angle deviation suggests that the model not only selects correct rotameric states at similar rates but also places sidechains closer to their experimentally observed conformations. These improvements are particularly relevant for applications that depend on fine-grained geometric accuracy, such as structure refinement, sidechain repacking, and protein design.

From a methodological perspective, our results demonstrate that modern machine learning techniques, when properly formulated with domain-appropriate inductive biases, can match or exceed the performance of carefully curated empirical libraries. The Dirichlet framework’s advantages include natural handling of uncertainty, smooth interpolation in sparse regions, and the principled treatment of periodic angular data, which all come at minimal computational cost while maintaining interpretability. In addition, a key advantage of the proposed framework is its ability to naturally incorporate additional contextual information. By extending the conditioning space to include a simple biochemical classification of nearby non-sequential residues, we observed small but consistent improvements in predictive performance without introducing instability or overfitting. This demonstrates that local environmental context contains meaningful information for sidechain modeling and that the Dirichlet formulation can exploit this information in a principled and scalable manner.

In summary, the proposed Dirichlet-based model offers a robust and extensible alternative to classical rotamer libraries. It preserves the interpretability and reliability of established methods while enabling smoother interpolation, improved geometric accuracy, and straightforward integration of additional contextual information. These properties make it a promising foundation for sidechain modeling approaches in structural biology and protein design.

## Figures and Tables

**Table 1:** The Dunbrack 2011 library performance.

AA	Set A			Set B	
	N ^1^	Acc. % ^2^	MADχ ^3^	Acc. %	MADχ
ARG	1099	35.46/33.76	49.27/46.49	35.18/35.60	49.31/45.79
ASN	1039	53.27/52.94	54.24/54.57	58.00/58.18	44.90/45.92
ASP	1366	65.01/64.86	33.97/33.71	66.42/64.48	32.08/33.39
CYS	278	69.06/67.63	40.55/42.63	68.46/67.80	41.29/41.96
GLN	908	37.41/38.25	60.21/55.24	37.68/39.72	56.72/51.18
GLU	1345	41.51/40.40	43.44/43.41	41.93/40.31	42.08/42.68
HIS	479	50.31/51.67	57.96/56.24	50.81/51.30	60.35/59.44
ILE	1320	76.70/76.83	27.38/27.97	77.92/77.39	26.51/26.63
LEU	2018	67.52/67.15	31.62/31.82	68.38/68.75	31.64/31.04
LYS	1272	39.58/38.86	42.34/42.33	41.00/39.45	40.56/41.18
MET	481	37.84/37.70	62.99/58.53	41.23/39.23	61.11/57.58
PHE	879	69.62/68.94	27.54/29.23	72.05/71.06	25.85/27.52
PRO	1011	75.17/80.42	20.92/24.78	78.87/82.83	16.75/23.19
SER	1442	59.22/58.74	53.67/54.11	59.37/58.34	53.75/54.89
THR	1349	79.39/78.43	30.10/31.31	81.47/80.56	27.13/28.02
TRP	330	54.39/47.27	56.34/61.62	53.70/50.97	57.04/58.64
TYR	844	70.6270.62	26.95/27.64	69.71/69.35	27.59/28.66
VAL	1531	86.35/86.48	13.89/13.85	86.61/86.76	13.13/13.10
Average		59.36/58.89	47.21/47.81	60.49/60.12	45.68/46.41

^1^ Number of occurrences of the amino acid in set A. ^2^ The accuracy of the amino acid for Dunbrack 2011/in-house’s Dunbrack 2011. ^3^ Mean absolute angular deviation (in degrees) for Dunbrack 2011/in-house’s Dunbrack 2011.

**Table 2:** Dirichlet method performance on backbone only.

AA	Set A		Set B	
	Acc. %	MADχ	Acc. %	MADχ
ARG	34.96	49.28	34.52	49.70
ASN	52.21	55.86	56.53	46.40
ASP	64.75	34.30	65.52	32.83
CYS	69.78	40.80	68.00	42.32
GLN	38.40	57.71	38.54	54.67
GLU	41.78	43.26	41.49	42.39
HIS	47.91	61.65	49.78	61.58
ILE	76.29	27.93	77.98	26.81
LEU	66.38	32.74	67.60	32.64
LYS	40.13	42.41	40.73	41.07
MET	40.40	60.50	40.91	60.48
PHE	69.23	29.12	71.60	26.78
PRO	76.41	21.01	79.83	16.52
SER	59.08	53.64	58.54	54.86
THR	79.32	30.53	80.86	28.10
TRP	50.91	59.10	52.32	57.99
TYR	69.67	28.04	69.24	28.64
VAL	86.41	14.10	86.88	13.67
Average	59.11	41.22	60.05	39.86

**Table 4:** Statistical validation of MADχ improvement.

Test Set	Method	MADχ (°)	ΔMADχ (°)	95%CI	t-stat.	*p*-val.	Cohen’s d
Set A	D-2011 *	47.21	-	-	-	-	-
(18.99 K)	Dirichlet	41.22	−6.0	[−6.8, −5.2]	14.73	<10^−45^	0.31
Set B	D−2011 *	45.68	-	-	-	-	-
(90 K)	Dirichlet	39.86	−5.8	[−6.1, −5.5]	33.04	<10^−220^	0.29

* D-2011 is the Dunbrack 2011. Negative ΔMADχ indicates improvement (lower error). *p* < 0.001.

**Table 5:** Sensitive analysis to alpha (α) and smoothing hyperparameters.

Radius	Sigma (σ)	Alpha (α)	Acc. %	MADχ
(1, 1, 1)	(1.0, 1.0, 1.0)	20	59.36	41.04
(1, 1, 1)	(1.0, 1.0, 1.0)	40	59.36	41.04
(1, 1, 1)	(1.0, 1.0, 1.0)	50	59.36	41.04
(1, 1, 1)	(1.0, 1.0, 1.0)	60	59.36	41.04
(1, 1, 1)	(1.0, 1.0, 1.0)	80	59.36	41.04
(1, 1, 1)	(1.0, 1.0, 1.0)	100	59.36	41.04
(1, 1, 2)	(4.0, 4.0, 2.0)	40	59.40	40.89
(1, 1, 2)	(4.0, 4.0, 2.0)	100	59.44	40.89
(0, 0, 0)	(1.0, 1.0, 1.0)	40	59.38	40.94
(2, 2, 2)	(1.0, 1.0, 1.0)	40	59.42	40.88
(1, 1, 1)	(1.5, 1.5, 1.5)	40	59.43	40.89
(1, 1, 1)	(2.0, 2.0, 2.0)	40	59.43	40.89

**Table 6:** The threshold was used and the number of clusters (#) for each amino acid (AA).

AA	RMSD Å	# Clusters	AA	RMSD Å	# Clusters
ARG	0.80	1061	LYS	0.70	339
ASN	0.70	47	MET	0.80	199
ASP	0.30	305	PHE	0.35	301
CYS	0.40	12	PRO	0.40	5
GLN	0.70	374	SER	0.35	12
GLU	0.90	123	THR	0.40	12
HIS	0.50	135	TRP	0.80	122
ILE	0.60	38	TYR	0.30	459
LEU	0.40	175	VAL	0.40	11

## Data Availability

The original contributions presented in this study are included in the article. Further inquiries can be directed to the corresponding author.
